# Improving medication adherence in the community: a purposive umbrella review of effective patient-directed interventions that are readily implementable in the United Kingdom National Health Service

**DOI:** 10.1007/s11096-025-01885-4

**Published:** 2025-03-14

**Authors:** Adam J. Mackridge, Eifiona M. Wood, Dyfrig A. Hughes

**Affiliations:** 1 Ysbyty Gwynedd, Betsi Cadwaladr University Health Board, Bangor, Gwynedd LL57 2PW UK; 2 Centre for Health Economics and Medicines Evaluation, Bangor University, Bangor, Gwynedd LL57 2PZ UK

**Keywords:** Medication adherence, Long-term conditions, Patient-focused interventions, Umbrella Review

## Abstract

**Background:**

Suboptimal medication adherence is a major determinant of treatment outcome. Between a third and a half of prescribed medicines for long-term conditions are not taken as intended, the reasons for which are numerous and multifaceted. Improving medication adherence should optimise therapeutic outcomes.

**Aim:**

To identify effective and readily implementable patient-focused interventions for improving medicines adherence that can inform best practice to improve health outcomes.

**Method:**

Medline, CINAHL and EMBASE were searched on 11 May 2022 for publications added since 11 January 2013, along with citation searches linked to Nieuwlaat’s 2014 Cochrane review. An umbrella review was undertaken of meta-analyses and systematic reviews of empirical research to identify and describe interventions that improve medication adherence. Effective interventions were assessed for their implementation potential.

**Results:**

Strategies to improve medication adherence follow common themes. Fifteen reviews and meta-analyses were identified, and interventions were grouped into eight types. These included using pharmacists to provide interventions; providing face to face interventions; using combination formulations; providing reminders and prompting mechanisms; giving feedback on individual adherence rates; promoting positive habits; using strategies to enhance self-management and positive behaviours; and using interventions in parallel.

**Conclusion:**

There are several readily implementable intervention approaches with demonstrable effectiveness based on systematic review or meta-analysis evidence. However, owing to the diverse evidence base in this field, and the significant risk of bias in many studies, further work is needed to understand the comparative value of different interventions and their impact on patient-oriented outcomes.

**Supplementary Information:**

The online version contains supplementary material available at 10.1007/s11096-025-01885-4.

## Impact statements

- Implementing effective medicines adherence interventions in NHS pharmacy settings offers potential to improve patient therapeutic outcomes.
- For maximum benefit, face to face, multicomponent approaches should be considered where possible.
- Community pharmacists should be commissioned to engage with patients and deliver effective interventions.

## Introduction

Sub-optimal medicines adherence can impair patients’ health-related quality-of-life, reduce life expectancy, and impact on healthcare services. The Organisation for Economic Co-operation and Development estimates that, annually, poor adherence contributes to nearly 200,000 premature deaths across Europe, costing €125 billion in excess healthcare provision [1].

The importance of designing strategies to enhance medication adherence through education-based, behavioural, and/or technological interventions is well established and some have theorised that improving adherence to existing treatments could have a greater impact on health outcomes than improvements to treatments would achieve [2]. Indeed, the World Health Organization declared medication adherence an issue of global importance, encouraging policy makers and health managers to improve public health through effective adherence support [3, 4].

Non-adherence manifests as failure to initiate therapy (not collecting prescribed medicines), variable implementation of therapy (irregular taking of treatment), and early discontinuation (failing to persist with treatment) [3, 5]. Reasons for non-adherence are numerous and multi-faceted; they can be intentional (due to health beliefs, attitudes to medicines, lack of recognition of treatment value, adverse effects, etc.), or unintentional (due to difficulty identifying medicines or following a dosing schedule, trouble with packaging/devices, memory issues etc.) [6–8]. Frequently, non-adherence involves an interaction between many determinants which evolve over time. Moreover, as patients take greater numbers of medicines, and live with more complex morbidities, challenges in remaining adherent, and implications of failing to do so, are increasing [9]. Owing to the underlying complexity of non-adherence there is a need to better understand which interventions have evidence of effectiveness in increasing adherence, so that practitioners can make meaningful interventions in patient care. Additionally, for realisation of population-level benefit, interventions need to be readily implementable within the appropriate care setting [10].

### Aim

To identify effective and readily implementable patient-focused interventions for improving medicines adherence that can inform best practice to improve health outcomes.

## Method

Taking a purposive approach, this umbrella review focused on meta-analyses and systematic reviews of existing empirical research [11], covering interventions with proven effectiveness in improving medication adherence across disease groups, populations and technology types. This review was conducted within the context of the National Health Service (NHS) in Wales but is considered applicable to the NHS across the UK as well as comparable healthcare service contexts. Medline, CINAHL and EMBASE, were searched on 11 May 2022. The search strategy was targeted to provide specificity relevant to the study topic; the search terms used for all databases were: “(adherence OR compliance OR non-adherence OR non-compliance) AND (medicine OR medicines OR medication OR drugs) AND (Interventions OR intervention)”, with limits of: Title Only; Review; after 11 Jan 2013 (given that research prior to this date was included in the Nieuwlaat et al. Cochrane review [12]); English language. PubMed and Scopus were also searched for citations of the 2014 Cochrane review by Nieuwlaat et al. [12].

Retrieved titles were screened by AJM. Abstracts of those considered potentially eligible were obtained and further screened. The full-text of articles included at this stage were then screened independently for inclusion and exclusion by AJM and EMW.

Papers were included if they: (1) were meta-analyses or PRISMA-reported systematic reviews [13] and (2) presented findings relating to patient-focused interventions with quantitative or qualitative measures of intervention effect on improved adherence.

Papers were excluded at full text sift if they: (1) focused on single diseases or on bespoke technologies, or (2) focused on healthcare systems or contexts that would not be transferable to the NHS. The references of all underlying studies in the included reviews were collated in an Excel spreadsheet and unique studies identified in a pivot table using DOI or PubMed ID or, where neither were available, article title. The Corrected Covered Area Index, a metric indicating the degree to which included reviews consist of the same or different primary research studies and returns a percentage overlap of primary studies, was then calculated [14].

Intervention details with statistically significant evidence of effect on adherence were themed by the nature of the intervention and risk of bias was also considered. Where possible, quantitative results were reported, otherwise a narration was used. For each intervention with a statistically significant effect, key findings were then summarised and categorised into implementable actions. Where moderator analysis was used to compare common components of interventions across multiple studies, effect size was reported using standard mean difference for the moderator analysis and is denoted as ‘MA SMD’ [15]. Review quality was assessed independently using the AMSTAR 2 instrument [16]. An independent assessment by three pharmacists (AJM, EMW, & DAH) considered the implementation potential of each recommended action based on acceptability, adoption, appropriateness, feasibility, fidelity, implementation cost, penetration, and sustainability, within the NHS context [17]. Confidence scores ranged from 1 (low) to 3 (high) for each criterion per adherence intervention. Concordance between assessors was measured using Kendall’s W coefficient of concordance for ranks [18].

## Results

### Effective adherence interventions

**Fig. 1:**
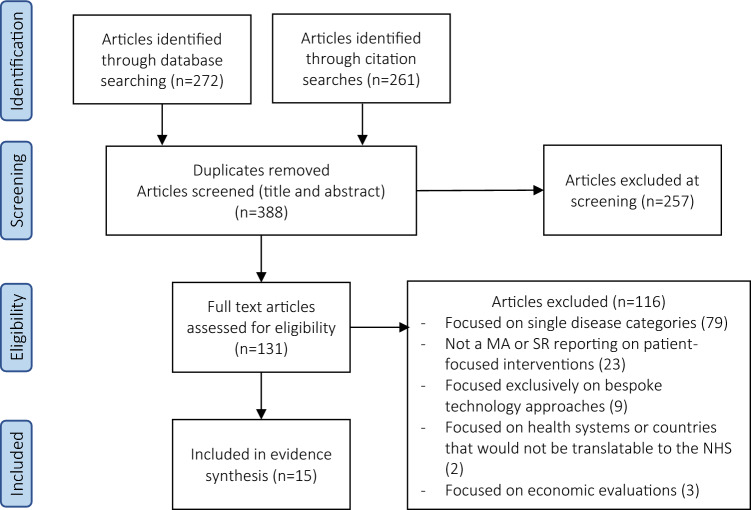
PRISMA Flowchart of article selection [13]

Fifteen articles met the inclusion and exclusion criteria (Fig. 1). Of these, eight included meta-analyses, one of which used Cochrane review methodology. Details of included reviews are given in Table 1. From these, 24 effective interventions were classified into eight implementable actions (Table 2), outlined below. The Corrected Covered Assessment identified an underlying overlap of 1.7% [14]. All fifteen articles were judged to be of “critically low” quality by the AMSTAR 2 instrument (Supplementary Appendix), principally because of a lack of an explicit statement that the review methods were established a priori, incomprehensive search strategies, limited detail of excluded studies, and for not accounting for risk of bias in individual studies when interpreting the results of the review.

**Table 1:** Details of included reviews

Paper	Review type	Aim(s) or research question(s)	Databases and search periods	Number of studies (Breakdown of included study/review types)
Conn and Ruppar 2017 [15]	SR + MA	What are the average effects of interventions on adherence? Do intervention effects vary depending on study design or sample characteristics? Do intervention effects vary depending on intervention characteristics?	MEDLINE, PsycINFO, CINAHL, EBSCO, PubMED, Cochrane Central Trials Register, Cochrane Database of Systematic Reviews, PQDT, ERIC, IndMed, International Pharmaceutical Abstracts, EBM Reviews—Database of Abstracts of Reviews of Effects, and Communication and Mass Media, plus nineteen research registries, conference abstracts from 48 conferences, and hand searches of 57 journals Search period: prior to 2015	771 (NR)
Milosavljevic et al. 2018 [19]	SR	What disease states and health issues are community pharmacist-led interventions targeting and what is the impact of these interventions on patients’ medication adherence and clinical, humanistic, economic and/or other health outcomes?	Medline, EMBASE, International Pharmaceutical Abstracts, ProQuest Dissertations and Theses and Google Scholar Search period: prior to October 2015	22 (16 RCTs; 6 non-RCT)
Conn et al. 2016 [20]	SR + MA	What are the overall standardised mean difference (SMD) outcomes for quality of life, knowledge, physical function, and symptoms after medication adherence interventions in a diverse sample of acutely and chronically ill patients? What are the SMDs for these outcomes when interventions are delivered to patients having specific types of illnesses such as diabetes or cardiovascular disease? What is the risk of bias in extant studies?	MEDLINE, PsycINFO, CINAHL, EBSCO, PubMED, Cochrane Central Trials Register, Cochrane Database of Systematic Reviews, PQDT, ERIC, IndMed, International Pharmaceutical Abstracts, EBM Reviews—Database of Abstracts of Reviews of Effects, and Communication and Mass Media, plus nineteen research registries, conference abstracts from 48 conferences, and hand searches of 57 journals Search period: prior to 2009	141 (NR) *Participants: 23,818 (12,715 intervention / 10,603 control)*
Baumgartner et al. 2020 [21]	SR	Examine the evidence for that idea and assesses the evidence of the effects of a reduced pill burden on medication adherence	PubMed, Web of Science, Cochrane Library, and Scopus Search period: between 1 Jan 2000 and 1 May 2019	84 (8 MA; 7 SR + MA; 2 SR;67 primary studies)
Tao et al. 2015 [22]	SR + MA	To conduct a meta-analysis of randomised controlled trials (RCTs) that assessed electronic reminders in chronic disease care and to determine their effects on patient adherence to medication	CINAHL Plus, MEDLINE, Cochrane Central Register of Controlled Trials and Web of Science Search period: prior to January 2014	22 (22 RCTs)
Yang et al. 2022 [23]	SR	To synthesise the evidence on the effectiveness of interventions to improve medication adherence in community-dwelling older people with multimorbidity to inform evidence-based strategies to promote adherence and health outcomes in this population	Airiti Library, China National Knowledge Infrastructure, Cochrane CENTRAL, EBSCO CINAHL, OVID EMBASE, OVID MEDLINE, Proquest Central, PsycINFO, Wanfang Database, and Web of Science Core Collection Search period: prior to October 2020	9 (5 RCT; 4 cRCT)
Thakkar et al. 2016 [24]	SR + MA	To estimate the effect of text messaging on medication adherence in adults with chronic medical disorders To describe and examine the effect of characteristics of text message interventions, including frequency of messaging, interactivity, and customisation To describe perceptions and acceptability to participants	MEDLINE, EMBASE, CINAHL, PsycINFO, Cochrane Central Register of Controlled Trials, and trial registries clinicaltrials.gov and anzctr.org.au Search period: prior to 15 January 2015	16 (16 RCTs)
Park et al. 2014 [25]	SR	To evaluate the efficacy of mobile phone interventions to improve medication adherence and to describe the characteristics of the interventions To explore acceptability and satisfaction of mobile phone interventions and to evaluate the selected studies in terms of study rigour, impact, cost and resource feasibility, generalisability and implications for nursing practice and research	PubMed, Web of Science, CINAHL, PsycInfo, Google Chrome and Cochrane databases Search period: between January 2002 and January 2013	29 (19 RCTs; 2 QES; 1 ROCS; 6 Pilots with no comparator group)
Pouls et al. 2021 [26]	SR	To evaluate effectiveness of recent interactive eHealth interventions on medication adherence in adult patients using long-term medication To describe applied strategies within effective interventions	MEDLINE, EMBASE, Cochrane Library, PsycINFO, and Web of Science Search period: between 2014 and July 2019	21 (21 RCTs)
Vervloet et al. 2012 [27]	SR	To synthesise and critically appraise the existing evidence on the effectiveness of electronic reminders in improving patients’ adherence to chronic medication To investigate the characteristics of electronic reminders that are associated with their effectiveness	PubMed, Embase, PsycINFO, CINAHL and the Cochrane Central Register of Controlled Trials Search period: prior to 7 March 2011	13 (13 RCTs)
Demonceau et al. 2013 [28]	SR + MA	To identify and to compare the efficacy of strategies and components thereof that improve implementation of the prescribed drug dosing regimen and maintain long-term persistence, based on quantitative evaluation of effect sizes across the aggregated trials	MEDLINE, EMBASE, CINAHL, the Cochrane Library, and PsycINFO Search period: prior to 31 December 2011	79 including in SR of which 48 were included in MA (74 RCT; 5 cRCT)
Seewoodharry et al. 2017 [29]	SR + MA	To explore whether feedback, guided by subjective or objective adherence measures, improves adherence	MEDLINE, Embase, CINAHL, and PsycINFO Search period: prior to 2016	24 including in the SR, of which 6 were including in the MA (24 RCTs)
Easthall et al. 2013 [30]	SR + MA	To describe and evaluate the use of cognitive-based behaviour change techniques as interventions to improve medication adherence	MEDLINE, EMBASE, PsychINFO, CINAHL and Cochrane databases Search period: prior to April 2013	26 (26 RCTs)
Cross et al. 2020 [31]	SR + MA (CR)	To evaluate the effectiveness of interventions designed to improve medication-taking ability and/or medication adherence in older community-dwelling adults prescribed multiple long-term medications	MEDLINE, Embase, Cochrane Central Register of Controlled Trials (CENTRAL), PsycINFO, CINAHL Plus, and International Pharmaceutical Abstracts Search period: prior to June 2019	50 (40 RCTs; 6 cRCTs; 4 qRCTs)
Viswanathan et al. 2012 [32]	SR	To assess the comparative effectiveness of patient, provider, systems, and policy interventions that aim to improve medication adherence for chronic health conditions in the United States	MEDLINE and the Cochrane Library Search period: prior to 4 June 2012	73 (69 RCTs; 4 OSs)

NR, not reported; MA, meta-analysis; SR, systematic review; CR, Cochrane review; RCT, randomised controlled trial; cRCT, cluster randomised controlled trial; QES, quasi-experimental study; ROCS, retrospective observational cohort study; qRCT, quasi randomised controlled trial; OS, observational study

**Table 2:** Effective adherence interventions from meta-analyses and systematic review findings, classified under eight recommended actions

Paper	Take home message	Effectiveness measure & comparator
*Use Pharmacists to provide interventions*		
Conn and Ruppar 2017 [15]	Interventions delivered by pharmacists were significantly more effective than those delivered by other health care professionals	Effect size (moderator analysis standardised mean difference): 0.337 (pharmacist delivered interventions) vs 0.279 (non-pharmacist delivered interventions) *p* = 0.031
Milosavljevic et al. 2018 [19]	Community pharmacist-led interventions have been shown to contribute to improved adherence and better disease control	61.5% of 65 outcomes across the 22 included studies showed a statistically significant result (*p* < 0.05) in favour of the intervention
*Deliver face-to-face interventions, ideally in a pharmacy setting*		
Conn and Ruppar 2017 [15]	Interventions delivered face-to-face were more effective than interventions delivered in other ways, such as by computer, telephone, surface mail, text messages, and written materials	Effect size (moderator analysis standardised mean difference): 0.331 (face-to-face delivery) vs 0.222 (non-face-to-face delivery) *p* < 0.001
	Interventions were less effective at improving adherence when they were delivered in subjects’ homes as compared to other locations such as clinics or pharmacies	Effect size (moderator analysis standardised mean difference): 0.315 (delivered outside of patients’ homes) vs 0.242 (delivered in patients’ homes) *p* = 0.006
	Interventions delivered by computer were significantly less effective than interventions delivered by other means	Effect size (moderator analysis standardised mean difference): 0.066 (interventions that included an element of computer-delivery) vs 0.295 (non-computer-delivered interventions *p* < 0.001
Conn et al. 2016 [20]	Face-to-face delivery is more effective than delivery through other mechanisms for patients with adherence difficulties	Effect size (moderator analysis standardised mean difference): 0.411 (face-to-face interventions) vs 0.182 (interventions delivered via mechanisms such as telephone or email) *p* = 0.050
*Use combination formulations to simplify dosing regimens*		
Baumgartner et al. 2020 [21]	Adherence can be improved through the use of combination formulations (polypills)	6 of 7 RCTs (86%) showed improved adherence with a combination formulation 31 of 39 high quality observational studies (79%) showed improved adherence with a combination formulation
*Provide patient reminders/prompting mechanisms (any type, including technology, text messages, apps)*		
Tao et al. 2015 [22]	Electronic reminders (i.e. SMS, alarm device and pager) were associated with a significant, yet small, improvement in patient adherence to medication, compared to non-use of reminders	Effect size (magnitude of difference in medication adherence, Cohen’s d): 0.29 (95% CI 0.18–0.41) for people receiving reminders vs those not receiving reminders
Yang et al. 2022 [23]	Electronic health interventions that provided medication-taking reminders, remote medication monitor and medication education for patients via electronic instruments such as computers and mobile phones demonstrated a small yet significant benefit for medication adherence	Effect size (moderator analysis standardised mean difference): 0.22 (95% CI 0.02–0.42; *p* = 0.03) for those receiving medication taking reminders, monitoring, or education
Thakkar et al. 2016 [24]	Mobile phone text messaging (incorporating text messages at a fixed predetermined frequency, real-time medication monitoring reminders where the medication dispenser had not been opened, and personalised messages) increases the odds of medication adherence	Odds Ratio 2.11 (95% CI 1.52–2.93 *p* < 0.001) for improved adherence amongst those receiving text message reminders vs those not receiving reminders
Park et al. 2014 [25]	Mobile phone text messaging may improve medication adherence	studies demonstrated statistically significant improvement in adherence or biomarkers (*p* < 0.05) following SMS interventions
Pouls et al. 2021 [26]	Simple eHealth technologies such as SMS text messaging, mobile apps, monitoring devices, interactive voice response, and telephone calls can be effective in promoting medication adherence in a wide variety of patient populations	Seventeen of twenty-nine of studies (59%) demonstrated a statistically significant improvement (*p* < 0.05) in medication adherence for those receiving an eHealth intervention
Vervloet et al. 2012 [27]	Electronic reminders via SMS, reminder devices and pagers can be effective in the short-term at improving adherence; however, long-term effects remain unclear	Improved adherence was found in three of four studies (75%) using SMS reminders, four of seven (57%) studies using electronic reminder devices and one of two (50%) using pager intervention
*Provide feedback on patient adherence, particularly in visual format*		
Demonceau et al. 2013 [28]	Electronically-monitored adherence feedback (EM-feedback) is a potentially effective approach to enhance patient adherence to medications	Effect size (percentage point difference in adherence): 19.8% (95% CI 10.7–28.9; interventions including EM-feedback) vs 10.3% (95% CI 7.5–13.1%; interventions not including EM-feedback) *p* < 0.01
Seewoodharry et al. 2017 [29]	Feedback guided by objective or subjective measures of adherence improves adherence	Effect size (increase in mean percentage adherence between baseline and follow-up for intervention; feedback provided vs control): 10.02% (95% CI 3.15–16.89; *p* = 0.004)
	Studies allowing visualisation of adherence data reported a higher improvement in adherence	In general, across 18 studies, adherence improved more in the group receiving visual feedback as part of their intervention and in 5 studies, this improvement exceeded a 10% improvement
*Use interventions that promote the formation of positive habits*		
Conn et al. 2016 [20]	Interventions that incorporated behavioural strategies such as prompts or cues to take medication (signs on fridge door, placing medicines where meals are eaten) were more effective than interventions that did not include prompts or cues	Effect size (moderator analysis standardised mean difference): 0.497 (interventions incorporating prompts or cues for taking medication) vs 0.234 (no prompts or cues) *p* = 0.034
	Habit-based interventions that examined the participants’ daily routines and then linked medication administration to those routines were more effective than interventions that did not focus on habits	Effect size (moderator analysis standardised mean difference): 0.574 (interventions promoting habit forming) vs 0.222 (no habit-forming component) *p* = 0.007
*Use strategies to enhance self-management and promote positive behaviour change*		
Demonceau et al. 2013 [28]	Cognitive-educational interventions which present information verbally, written or audio-visually to educate and motivate patients so that they more empowered and more likely to adhere are potentially effective approaches to enhance patient adherence to medications	Effect size (percentage point difference in adherence): 16.1% (95% CI 10.7–21.6%) for those receiving cognitive-educational interventions vs 10.1% (95% CI 6.6–13.6%) for those receiving interventions without cognitive-educational components *p* = 0.04
Easthall et al. 2013 [30]	Cognitive-based behaviour change techniques such as motivational interviewing are associated with small but statistically significant improvements in medication adherence	Effect size (standardised difference in means, Hedges’ g): 0.34 (95% CI 0.23 to 0.46) for those receiving interventions incorporating cognitive-based behaviour change techniques vs those receiving interventions that did not included these; *p* < 0.001
Yang et al. 2022 [23]	Interventions to improve self-management may have a significant but modest improvement to medication adherence	Effect size (standardised mean difference): 0.52 (95% CI 0.04–0.99) for those receiving interventions that focused on self-management techniques vs those receiving interventions that did not include this component; *p* = 0.03
Pouls et al. 2021 [26]	Intervention strategies that focus on improving patients’ involvement in their treatment, alongside improving their medication management skills are most promising	Narrative finding: 23 of 29 (79%) studies involved interventions that informed and educated patients about their treatment, whilst 15/29 (52%) focused on providing assistance and encouragement
*Use multicomponent approaches*		
Cross et al. 2020 [31]	Mixed interventions involving both educational and behavioural components may improve the proportion of people who are adherent	Risk ratio = 1.22 (95% CI 1.08 to 1.37) for those receiving mixed educational and behavioural interventions in comparison to those not receiving these interventions
Viswanathan et al. 2012 [32]	Education with behavioural support enhances adherence	Narrative finding that mixed education with behavioral support was more effective than single intervention types

MA, meta-analysis; SR, systematic review; CR, Cochrane review

#### Use pharmacists to provide interventions

Two reviews reported the benefits of engaging pharmacists in medication adherence activities. A meta-analysis of 771 studies reported that pharmacist-delivered interventions were more effective in improving adherence than interventions delivered by others (MA SMD effect size 0.337 vs 0.279, *p* = 0.031 [15]). Similarly, a systematic review including patients who received interventions delivered by community pharmacists demonstrated statistically significant improvement (*p* < 0.05) in adherence in 61.5% of 65 outcomes, although it was not clear against which comparators these interventions were tested [19].

#### Deliver face-to-face interventions, ideally in a pharmacy setting

Two reviews provided evidence on the importance of selecting the appropriate mode of delivery. Interventions delivered face-to-face are more effective than non-face-to-face methods (MA SMD effect size: 0.411 vs 0.182; *p* = 0.050 [20]; 0.331 vs 0.222; *p* < 0.001 [15]) and interventions that include some form of computer delivery are less effective than interventions that do not include computer delivery (MA SMD effect size: 0.066 vs 0.295; *p* < 0.001 [15]). Face-to-face consultations held outside of patients’ homes, such as in a pharmacy, are also preferable to delivery in patients’ homes (MA SMD effect size: 0.315 vs 0.242; *p* = 0.006 [15]).

#### Use combination formulations to simplify dosing regimens

Baumgartner et al. found that fixed dose combination formulations, ‘polypills’, improved adherence in 6 out of 7 RCTs, further supported by evidence from 31 of 39 observational studies, which were judged as high quality by the review authors [21].

#### Provide patient reminders/prompting mechanisms

Tao et al. reported that SMS or alarm/pager devices were associated with a small yet significant improvement in adherence versus standard care (Cohen’s d 0.29; 95% CI 0.18–0.41 [22]) with similar findings observed in a meta-analysis (SMD 0.22; 95% CI 0.02–0.42; *p* = 0.03 [23]). Thakkar et al. also reported that text message interventions, including personalised messages, significantly improved medication adherence versus standard care (Odds Ratio 2.11, 95% CI 1.52–2.93; *p* < 0.001 [24]). Several literature reviews support this finding, including Park et al., where 18 of 29 studies demonstrated statistically significant improvement in adherence rates or biomarkers when SMS interventions were provided [25]. Pouls et al. reported that simple eHealth interventions which incorporated reminders to take medication demonstrated a statistically significant improvement in medication adherence in 59% of interventions [26]. Vervloet et al. also found that adherence improved for patients in three of four studies using SMS reminders, four of seven using electronic reminder devices and one of two using pager reminders [27].

#### Provide feedback on patient adherence, particularly in visual format

Demonceau et al. showed that interventions which include feedback from electronically monitored medication events were more effective at improving adherence than interventions without (% adherence: intervention vs control 19.8% (95% CI 10.7–28.9) vs 10.3% (95% CI 7.5–13.1); *p* < 0.01 [28]). In a meta-analysis of six studies, mean percentage adherence improved by 10.02% (95% CI 3.15–16.89; *p* = 0.004) more between baseline and follow-up when both objective and subjective monitoring of adherence were applied and that in 5 of 18 studies a general enhanced benefit was seen with a relative improvement over 10% when visual feedback of adherence was provided [29].

#### Use interventions that promote the formation of positive habits

Conn et al. reported that promoting the use of prompts or cues is effective in comparison to not providing prompts or cues (MA SMD 0.497 vs 0.234; *p* = 0.034) and that habit-based interventions are also effective when compared to those that do not have a habit-forming component (MA SMD 0.574 vs 0.222; *p* = 0.007 [20]).

#### Use strategies to enhance self-management and promote positive behaviour change

Strategies that incorporate cognitive-educational components to educate and motivate patients with verbal, written, or audio-visual components improve adherence compared to those without cognitive-educational components (% difference in adherence: 16.1% vs 10.1% *p* = 0.04 [28]). Motivational interviewing also improved adherence (Hedges’ g 0.34; 95% CI 0.23–0.46; *p* < 0.001 [30]), as did interventions targeting self-management (SMD 0.52; 95% CI 0.04–0.99; *p* = 0.03 [23]). Intervention strategies to improve patients’ involvement in their treatment, alongside improving medication management skills are most promising [26].

#### Use multicomponent approaches

Whilst each of the above actions is, independently, likely to improve adherence, Cross et al*.* found that mixed interventions involving both educational and behavioural components improved the proportion of adherent people versus usual care (risk ratio = 1.22; 95% CI 1.08 to 1.37 [31]), whilst in a systematic review, Viswanathan et al. reported providing behavioural support alongside educational counselling enhanced adherence [32].

#### Implementation potential

Interventions identified as most promising for ease of implementation were (a) using pharmacists to provide interventions, (b) providing face-to-face interventions ideally in a pharmacy setting, and (c) using combination formulations to simplify dosing regimens (Table 3). Concordance amongst raters for each criterion per intervention was fair (Kendall’s W = 0.594; *p* = 0.0001).

**Table 3:** Implementation potential of adherence interventions—subjective assessment*

	Use Pharmacists to provide interventions	Provide face to face interventions, ideally in a pharmacy setting	Use combination formulations to simplify dosing regimens	Provide patient reminders/ prompting mechanisms (any type)	Provide feedback on patient adherence, particularly in visual format	Use interventions that promote the formation of positive habits	Use strategies to enhance self-management and promote positive behaviour change	Use multicomponent approaches
Acceptability	3.0	3.0	3.0	2.7	2.7	2.3	2.3	2.7
Adoption	3.0	2.7	2.0	2.0	2.0	2.3	2.0	1.7
Appropriateness	3.0	3.0	3.0	3.0	3.0	3.0	2.7	3.0
Feasibility	3.0	3.0	2.0	2.0	1.3	2.0	1.7	2.0
Fidelity	2.7	2.7	2.7	2.3	2.0	2.0	2.0	2.0
Implementation cost	1.7	1.7	2.3	1.7	1.3	2.0	1.0	1.0
Penetration	3.0	3.0	2.7	2.3	1.7	1.7	1.7	1.7
Sustainability	2.7	2.7	3.0	2.3	1.7	2.3	1.3	2.0
Sum of scores	22.0	21.7	20.7	18.3	15.7	17.7	14.7	16.0

^*^ Scores are means of each author’s independent assessment, ranging from 1 = low confidence to 3 = high confidence in each adherence intervention meeting implementation criteria relevant to the NHS setting [15]

## Discussion

### Key findings

A series of eight evidence-based recommendations that would be widely implementable within the NHS context were formulated. Pharmacists, already highly practised in informing patients about their medicines, are well placed to provide interventions likely to optimise adherence, particularly when multiple medicines and complex regimens are involved. Interventions are more effective if delivered face-to-face and in an out-of-home setting and community pharmacies, particularly with widespread availability of private consultation areas, provide an ideal location for such interventions.

Other reviews have identified polypharmacy as a cause of non-adherence [33], and combination formulations of multiple medicines, ‘polypills’, can simplify regimens to help improve adherence over multiple individual dosage forms. The usefulness of combination formulations is limited by the availability, and potentially cost, of suitable products; although in the latter case, the therapeutic benefits gained from improved adherence could justify increased costs, particularly where complex regimens are needed for successful management [34, 35]. In the UK, changes to prescribed formulations presently require the input of a prescriber, which may limit pharmacist roles in implementing change. However, UK legislative changes to allow all newly qualified pharmacists from 2026 to prescribe from the point of registration, as well as the ongoing training option for existing pharmacists to become independent prescribers, will remove this constraint [36–38].

Reminders or prompts to take medicines through text messages, smartphone applications (apps), or standalone devices are effective, as is providing feedback on individual patient adherence from, for example, smartphone app data. These may use approaches from automated reminders per dose, to daily, or periodic messages and using text messages via apps or built-in functionality for smartphones, or through specific adherence support devices [39–42]. Most evidence supports the use of SMS or pager devices and whilst pager devices have largely been superseded, SMS is effectively an equivalent technology.

Initial set up of a text messaging reminder infrastructure may be costly, but app or device-based interventions are already available at low cost and could likely be quickly implemented. Estimates suggest that up to 69% of over-65 s use a smartphone in the UK, indicating potential for wide reach [43]. Regardless of the technology, it is likely that some support would be needed to help patients to set up and/or use it, to minimise risk of medicines-related harm from incorrect operation.

Many apps can capture data on the response of patients giving the opportunity to collate and provide feedback on individual adherence [41]. This could be particularly useful for time-dependent dosing, such as in Parkinson’s Disease, or for weekly or atypical timings, which might be easily forgotten, such as for methotrexate, or bisphosphonates [44].

Supporting patients to form positive habits by identifying potential cues relevant to medicines use (e.g., sitting down for breakfast, or placing medicines where meals are eaten) would likely be easily implemented at low-cost. Pharmacists can be trained in effective techniques to promote the formation of positive habits though identifying potential triggers, where even small changes can build lasting habits [28, 29].

Strategies to enhance self-management and promote positive behaviour change are feasible in the NHS context and behaviour change interventions can effectively be delivered by community pharmacists for public health [45–47]. However, these are largely based on public health interventions and the learning from this may not be translatable to other contexts. To maximise benefit from this approach, further training in Motivational Interviewing may be beneficial, allowing adaptation of messages to align with the patient’s personal adherence challenges [48].

Whilst each of the above actions is likely to improve adherence, compelling evidence shows that using multiple approaches, tailored to individual patients and addressing the underlying causes of non-adherence, provides the greatest impact [49]. This approach enables patients to receive relevant and practical support, designed for their unique needs and could be achieved via the design and implementation of pharmacist-led patient-focused services to support adherence, delivered in a community pharmacy setting.

The rating exercise conducted by the authors to assess the implementation potential of each recommended action identified those interventions with greatest ease of implementation. These included using pharmacists, ideally for face-to-face consultations in the pharmacy setting and adopting combination formulations where possible. Other interventions, such as providing feedback on adherence, promoting behavioural change and using multicomponent approaches pose greater challenges since successful delivery would require investment in infrastructure and training in addition to pharmacist capacity.

### Strengths and weaknesses

This targeted umbrella review aimed to identify effective and readily implementable patient-focused interventions for improving medicines adherence that can inform best practice to improve health outcomes.

Review titles were screened and relevant abstracts reviewed by one investigator with a second investigator reviewing the full-text articles only which may have led to some reviews being inappropriately excluded. Reviews that reported no effect, or a negative effect, were not included, given our focus on plausibly effective interventions. By using meta-analysis and systematic review findings only, it is likely that the interventions identified as demonstrating an effect are those that have greatest promise for guiding practice development.

There is a very large body of research in this field [50] but many published studies are small, and/or at moderate to high risk of bias. A wide range of interventions have been tested and their impact on medication adherence is heterogeneous, so collation of findings to determine which interventions are effective is challenging [51]. Although most studies included in the included reviews were randomised controlled trials, a small number were observational studies. Across all the included studies, the risk of bias was highlighted by the authors. Sources of bias included publication bias, lack of blinding and randomisation within study designs, large numbers of small-scale studies, and lack of intention to treat models of analysis. It is acknowledged that the bias reported is based on the included reviews authors’ judgments and assessments rather than our independent bias assessment. A further potential source of bias arises from the poor quality of the included systematic reviews and meta-analyses. According to the AMSTAR 2 rating, reviews of “critically low quality” should not be relied on to provide an accurate and comprehensive summary of the available studies [16]. However, others have commented on the poor discrimination capacity of AMSTAR 2 [52], highlighted also by two Cochrane-standard reviews [24, 31] in our study being judged as critically low quality.

This purposive review sought to identify medication adherence interventions that could be easily implemented with the context of the NHS in Wales. Consequently, a pragmatic approach was undertaken and included reviews were restricted to those that were published in English and reported interventions that would likely be applicable and implementable within the NHS Wales community pharmacy setting. We do however believe that whilst this review has wider applicability within the NHS in the UK, as well as to other countries that employ similar healthcare delivery approaches, it may be less applicable in other health systems.

### Interpretation

Because of the evidence context, this review has attempted to identify the characteristics of interventions where the greatest benefit has been observed in larger randomised controlled trials however supporting data from observational studies may also be included. This has yielded a collection of recommendations that are reasonably likely to improve medication adherence within the existing frameworks of the NHS. Whilst an attempt at considering the implementation potential of each intervention has been made, this subjective assessment would need confirmation by further work exploring implementation within the NHS context. However, it is important to recognise that it is not currently possible to draw definitive conclusions from the available published data as to whether improved adherence translated to patient benefit and, whilst these findings are appropriate to guide practice development within the community pharmacy setting, care should be taken during implementation.

### Further research

Despite the extensive research in this area, the quality and scale of studies is frequently limited and more robust and well-designed studies using standardised interventions and outcome measures are needed to definitively identify the most effective interventions to support medication adherence in a real-world setting and support future policy and practice.

## Conclusion

Although the evidence-base for interventions that improve adherence is very large, it is significantly limited by risk of bias, and it is difficult to draw definitive conclusions relating to which interventions are most effective. However, this purposive umbrella review has identified eight actions that can realistically be implemented into practice, all of which have evidence from multiple sources demonstrating efficacy.

The most feasible mechanism to implement change within the NHS context in the short to medium term would be through commissioned services delivered through community pharmacies. This would enable actions to be implemented using multi-component approaches that are aligned to current NHS strategies for improvement.

## Supplementary Information

Below is the link to the electronic supplementary material.

Supplementary file1 (DOCX 29 KB)
