# Supplementary material for: Improving medication adherence in the community: a purposive umbrella review of effective patient-directed interventions that are readily implementable in the United Kingdom National Health Service
Source: Int J Clin Pharm. 2025 Mar 14;47(3):640–53. doi: 10.1007/s11096-025-01885-4 (PMC12125107; doi:10.1007/s11096-025-01885-4)
Supplement: Supplementary file 1 — Supplementary file1 (DOCX 29 KB) [file 11096_2025_1885_MOESM1_ESM.docx]

**Supplementary Appendix**

Quality assessment of included systematic reviews and meta-analyses, based on the AMSTAR 2 (A MeaSurement Tool to Assess systematic Reviews) instrument.

| **Item**  **Reference** | **1** | **2** | **3** | **4** | **5** | **6** | **7** | **8** | **9** |
| --- | --- | --- | --- | --- | --- | --- | --- | --- | --- |
| Conn & Ruppar 2017 [15] | No | No | No | No | No | Yes | No | No | RCTs: Partial Yes; NRS: No |
| Milosavljevic at al. 2018 [19] | No | No | No | Yes | Yes | No | No | No | RCTs: Yes; NRS: Yes |
| Conn et al. 2016 [20] | Yes | No | No | Yes | Yes | Yes | No | Partial Yes | RCTs: Partial Yes; NRS: No |
| Baumgartner et al. 2020 [21] | Yes | No | No | No | Yes | No | No | Yes | RCTs: Yes; NRS: Partial Yes |
| Tao et al. 2015 [22] | No | No | No | No | Yes | Yes | No | No | RCTs: Partial Yes; NRS: Includes only RCTs |
| Yang et al. 2021 [23] | Yes | Yes | No | No | Yes | Yes | No | Yes | RCTs: Yes; NRS: No |
| Thakkar et al. 2016 [24] | No | No | No | Yes | Yes | No | No | No | RCTs: Yes; NRS: Includes only RCTs |
| Park et al. 2014 [25] | Yes | No | Yes | No | No | No | No | Partial Yes | RCTs: Yes; NRS: No |
| Pouls et al. 2021 [26] | Yes | Yes | No | No | Yes | No | No | No | RCTs: Yes; NRS: Includes only RCTs |
| Vervloet et al. 2012 [27] | No | No | No | No | Yes | No | Yes | Partial Yes | RCTs: Yes; NRS: Includes only RCTs |
| Demonceau et al. 2013 [28] | Yes | No | No | No | Yes | No | No | No | RCTs: Yes; NRS: Includes only RCTs |
| Seewoodharry et al. 2017 [29] | No | No | No | No | Yes | Yes | No | No | RCTs: Partial Yes; NRS: Includes only RCTs |
| Easthall et al. 2013 [30] | No | Yes | No | Yes | Yes | Yes | No | No | RCTs: Yes; NRS: Includes only RCTs |
| Cross et al. 2020 [31] | Yes | No | No | Partial yes | Yes | Yes | Yes | Partial Yes | RCTs: Yes; NRS: Includes only RCTs |
| Viswanthan et al. 2012 [32] | No | Yes | Yes | No | Yes | Yes | No | No | RCTs: Yes; NRS: Yes |

| **Item**  **Reference** | **10** | **11** | **12** | **13** | **14** | **15** | **16** | **Overall quality** |
| --- | --- | --- | --- | --- | --- | --- | --- | --- |
| Conn & Ruppar 2017 [15] | No | RCTs: Yes; NRS: Yes | Yes | No | Yes | No | Yes | Critically low quality |
| Milosavljevic at al. 2018 [19] | No | No MA conducted | No MA conducted | No | No | No MA conducted | Yes | Critically low quality |
| Conn et al. 2016 [20] | No | RCTs: Yes; NRS: Yes | Yes | Yes | Yes | Yes | Yes | Critically low quality |
| Baumgartner et al. 2020 [21] | No | No MA conducted | No MA conducted | No | No | No MA conducted | Yes | Critically low quality |
| Tao et al. 2015 [22] | No | RCTs: Yes; NRS: Only RCTs | No | No | Yes | No | No | Critically low quality |
| Yang et al. 2021 [23] | No | RCTs: Yes; NRS: No | No | No | No | No | Yes | Critically low quality |
| Thakkar et al. 2016 [24] | No | RCTs: Yes; NRS: Only RCTs | Yes | No | Yes | Yes | Yes | Critically low quality |
| Park et al. 2014 [25] | No | No MA conducted | No MA conducted | No | No | No MA conducted | No | Critically low quality |
| Pouls et al. 2021 [26] | No | No MA conducted | No MA conducted | No | No | No MA conducted | Yes | Critically low quality |
| Vervloet et al. 2012 [27] | No | No MA conducted | No MA conducted | No | No | No MA conducted | Yes | Critically low quality |
| Demonceau et al. 2013 [28] | No | RCTs: Yes; NRS: Only RCTs | No | No | No | Yes | Yes | Critically low quality |
| Seewoodharry et al. 2017 [29] | No | RCTs: Yes; NRS: Only RCTs | No | No | Yes | Yes | Yes | Critically low quality |
| Easthall et al. 2013 [30] | No | RCTs: Yes; NRS: Only RCTs | No | No | Yes | Yes | Yes | Critically low quality |
| Cross et al. 2020 [31] | Yes | RCTs: Yes; NRS: Only RCTs | No | No | Yes | No | Yes | Critically low quality |
| Viswanthan et al. 2012 [32] | No | No MA conducted | No MA conducted | No | No | No MA conducted | Yes | Critically low quality |

Abbreviations:

MA Meta-analysis; RCT Randomised controlled trial; NRSI Non-randomised studies; PICO Population intervention comparison outcome

Items:

1. Did the research questions and inclusion criteria for the review include the components of PICO?
2. Did the report of the review contain an explicit statement that the review methods were established prior to the conduct of the review and did the report justify any significant deviations from the protocol?
3. Did the review authors explain their selection of the study designs for inclusion in the review?
4. Did the review authors use a comprehensive literature search strategy?
5. Did the review authors perform study selection in duplicate?
6. Did the review authors perform data extraction in duplicate?
7. Did the review authors provide a list of excluded studies and justify the exclusions?
8. Did the review authors describe the included studies in adequate detail?
9. Did the review authors use a satisfactory technique for assessing the risk of bias in individual studies that were included in the review?
10. Did the review authors report on the sources of funding for the studies included in the review?
11. If meta-analysis was performed did the review authors use appropriate methods for statistical combination of results?
12. If meta-analysis was performed, did the review authors assess the potential impact of risk of bias in individual studies on the results of the meta-analysis or other evidence synthesis?
13. Did the review authors account for risk of bias in individual studies when interpreting/ discussing the results of the review?
14. Did the review authors provide a satisfactory explanation for, and discussion of, any heterogeneity observed in the results of the review?
15. If they performed quantitative synthesis did the review authors carry out an adequate investigation of publication bias (small study bias) and discuss its likely impact on the results of the review?
16. Did the review authors report any potential sources of conflict of interest, including any funding they received for conducting the review?
